# Possible mechanisms underlying the association between human T-cell leukemia virus type 1 (HTLV-1) and hypertension in elderly Japanese population

**DOI:** 10.1186/s12199-021-00938-0

**Published:** 2021-01-29

**Authors:** Yuji Shimizu, Kazuhiko Arima, Yuko Noguchi, Shin-Ya Kawashiri, Hirotomo Yamanashi, Mami Tamai, Yasuhiro Nagata, Takahiro Maeda

**Affiliations:** 1 Department of Community Medicine, Nagasaki University Graduate School of Biomedical Sciences, Nagasaki-shi, Sakamoto 1-12-4, Nagasaki, 852-8523 Japan; 2 Department of Cardiovascular Disease Prevention, Osaka Center for Cancer and Cardiovascular Disease Prevention, Osaka, Japan; 3 Department of Public Health, Nagasaki University Graduate School of Biomedical Sciences, Nagasaki, Japan; 4 Department of General Medicine, Nagasaki University Graduate School of Biomedical Sciences, Nagasaki, Japan; 5 Department of Island and Community Medicine, Nagasaki University Graduate School of Biomedical Sciences, Nagasaki, Japan; 6 Center for Comprehensive Community Care Education, Nagasaki University Graduate School of Biomedical Sciences, Nagasaki, Japan

**Keywords:** Aging, Atherosclerosis, HTLV-1, Hypertension, Platelet

## Abstract

**Background:**

Human T-cell leukemia virus type 1 (HTLV-1) activates inflammatory cascades by activating the NF-κB pathway. The minor allele of single nucleotide polymorphism (SNP) in breast cancer suppressor BRCA1**-**associated protein (BRAP), which has a common etiology with HTLV-1 infection, has been reported to be positively associated with carotid atherosclerosis, but inversely associated with hypertension. Therefore, HTLV-1 infection may be inversely associated with hypertension by activating endothelial maintenance, including atherosclerosis. To clarify these associations, a cross-sectional study was conducted using 2989 Japanese individuals aged 60–99 years participating in a general health check-up.

**Methods:**

Logistic regression models were used to clarify the association between HTLV-1 and hypertension. Platelet levels stratified analyses were also performed since platelet production, which plays a crucial role in endothelium maintenance, can be stimulated by activating the NF-κB pathway.

**Results:**

HTLV-1 infection was found to be significantly inversely associated with hypertension, particularly in subjects with high platelet levels (≥ second tertiles of platelet levels); the fully adjusted odds ratios (ORs) and 95% confidence intervals (CIs) were 0.75 (0.62, 0.92) for total and 0.64 (0.50, 0.82) for high platelet levels, respectively. Further analysis of the non-hypertensive subjects demonstrated that HTLV-1 infection was significantly positively associated with atherosclerosis in subjects with the highest tertile of platelet levels (2.11 [1.15, 3.86]) but not in subjects with low platelet levels (first and second tertiles of platelet level) (0.89 [0.57, 1.39]).

**Conclusion:**

Asymptomatic HTLV-1 infection is inversely associated with hypertension, possibly by activating endothelial maintenance, including atherosclerosis progression.

## Background

Although human T-cell leukemia virus type 1 (HTLV-1), which is the earliest recognized human retrovirus, has been shown to induce adult T-cell leukemia/lymphoma [1], myelopathy/tropical spastic paraparesis, sensorimotor polyneuropathy, and optic neuritis [2], the majority of carriers remain asymptomatic throughout their lives [3–5]. However, asymptomatic HTLV-1 infection has been found to be positively associated with advanced periodontitis [6, 7], which is reported to be positively associated with atherosclerosis [8], platelet count, and inflammation [9]. Since HTLV-1 possesses a characteristic of enhancing inflammation [10, 11] possibly by activating the NF-κB pathway [12], its biochemical characteristics might have an influence on endothelial maintenance, including hypertension and atherosclerosis progression.

Single nucleotide polymorphisms (SNPs) in breast cancer suppressor BRCA1**-**associated protein (BRAP) activate an inflammatory cascade via the activation of the NF-κB pathway [13] and increase the risk of carotid atherosclerosis [14], while reducing the risk of hypertension [15, 16]. A minor allele of BRAP-related SNP (rs3782886) has been found to be positively associated with platelet count and inversely associated with hypertension [17, 18].

Therefore, HTLV-1 infection possesses common biochemical characteristics that promote the NF-κB pathway [12] with BRAP-related SNPs. Since activation of the NF-κB pathway promotes the production of platelet activation proteins [19], HTLV-1 infection could be inversely associated with hypertension by activating platelet production, resulting in the progression of atherosclerosis.

To evaluate this relationship, we conducted a cross-sectional study of 2989 elderly Japanese individuals (aged 60–99 years) who had previously participated in an annual health check-up between 2016 and 2018.

## Material and methods

### Study population

Considering the shortage of staff to conduct a health check-up in the present survey, the entire city population could not be surveyed in a span of 1 year. Therefore, we conducted the survey in different parts of Goto city over a period of 3 years to ensure that all the areas were covered.

The study population was comprised of 3013 individuals (1166 men and 1847 women) aged between 60 and 99 years from Goto City (western Japan) who had previously attended an annual health check-up conducted by the local government and directed by the Ministry of Health, Labor and Welfare in Japan during 2016–2018.

Participants without data on HTLV-1 (*n* = 14) or laboratory data (*n* = 10) were excluded. The remaining participants, comprised of 2989 elderly Japanese individuals (1152 men and 1837 women) with a mean age of 73.0 (standard deviation [SD], 7.4) for men and 73.0 (SD, 7.3) for women, were enrolled in the study.

Written consent forms were made available to ensure that the participants understood the objective of the study. Informed consent was obtained from all the participants. This study was approved by the Ethics Committee of Nagasaki University Graduate School of Biomedical Sciences (project registration no. 14051404).

### Data collection and laboratory measurements

The experimental protocols were reviewed by the medical staff in meetings before conducting the study to reduce inter-observer variability in the measurements.

Trained interviewers were tasked with obtaining the medical history of the participants and their habitual status (drinking and smoking). Body weight and height with bare feet and light clothes were measured using an automatic body composition analyzer (BF-220; Tanita, Tokyo, Japan). Body mass index (BMI) was calculated as weight (kg)/height (m)^2^.

After at least 5 min of rest, blood pressure (systolic and diastolic) was measured in the sitting position using a blood pressure measuring device (HEM-907; Omron, Kyoto, Japan). If the participants exhibited high levels of blood pressure at first (SBP ≥ 140 mmHg and/or DBP ≥ 90 mmHg), we also measured blood pressure again (second time), and then, the lower blood pressure values were used. Hypertension was defined as SBP ≥ 140 mmHg and/or DBP ≥ 90 mmHg and/or taking anti-hypertensive medication.

Blood samples were collected in a siliconized tube, a sodium fluoride tube, and an EDTA-2K tube. All measurements were obtained following the standard laboratory procedures at SRL, Inc. (Tokyo, Japan). The blood samples in the siliconized tube were used to measure high-density lipoprotein (HDL)-cholesterol and triglycerides and γ-glutamyltransferase (γ-GTP). The samples in the sodium fluoride tube were used to measure the levels of *glycated hemoglobin* (HbA1c). The quantities of white blood cells and platelets were determined using the blood in the EDTA-2K tube.

### Measurement of carotid intima-media thickness (CIMT)

Experienced vascular technicians measured carotid intima-media thickness (CIMT) using a LOGIQ Book XP with a 10-MHz transducer (GE Healthcare, Milwaukee, WI, USA). The maximum values for the left and right common carotid arteries of the CIMT were calculated using an automated digital edge-detection software (Intimascope; MediaCross, Tokyo, Japan) according to a previously described protocol [20]. The higher values of the right and left CIMT, not including plaque measurements, were then calculated. The maximum CIMT value was used for analysis. Since a previous study reported a normal CIMT value as < 1.1 mm [21], we defined atherosclerosis as a CIMT value of ≥ 1.1 mm.

### Measurement of human T-cell leukemia virus type-1 (HTLV-1)

To detect HTLV-1, a chemiluminescent enzyme immunoassay (CLEIA) kit (Fujirebio Inc., Tokyo, Japan) was used at SRL, Inc. (Tokyo, Japan).

### Statistical analysis

The characteristics of the study population in relation to the status of HTLV-1 infection are expressed as the mean ± SD for continuous variables, except for γ-GTP, prevalence of gender status (men), and medication status. Since γ-GTP showed a skewed distribution, the characteristics of the study population are expressed as the median [first quartile and third quartile], followed by logarithmic transformation. A trend test was performed using a regression model for the mean values.

Logistic regression models were used to calculate the odds ratios (ORs) and 95% confidence intervals (CIs) to determine associations between HTLV-1 and hypertension, the HTLV-1 infection status specific association between platelet and hypertension, and the category of platelet levels specific to the association between HTLV-1 and hypertension.

To emphasize the beneficial influence on the prevention of hypertension, further analysis was performed using subjects without hypertension.

Using logistic regression models, we analyzed the participants without hypertension and evaluated the specific association of HTLV-1 infection status between platelet count and atherosclerosis as well as the specific association for the category of platelet levels between HTLV-1 and atherosclerosis.

For all of the subjects, we evaluated the effect of HTLV-1 infection on the association between platelet and hypertension as well as the effect of platelet categories on the association between HTLV-1 infection and hypertension by logistic regression analysis. For subjects without hypertension, we evaluated the effect of HTLV-1 infection on the association between platelet count and atherosclerosis as well as the effect of platelet categories on the association between HTLV-1 infection and atherosclerosis by logistic regression analysis.

Two different models were used to adjust for confounding factors. Model 1 was adjusted for age and sex. For model 2, we included other potential confounding factors, including BMI (kg/m^2^), HDL-cholesterol (mg/dL), triglycerides (mg/dL), HbA1c (%), white blood cell (cells/μL), and γ-GTP (U/L). Smoking status and drinking status are known cardiovascular risk factors. However, these factors were not taken into account as they did not influence the endothelium directory; those factors induced physical stress on the endothelium by activating inflammation [22] and increasing oxidative stress [23, 24] but not indicate intravascular environment directory. Instead of using smoking or drinking status, we used the white blood cell count [22] and γ-GTP [23], since these factors directly affect the status of the blood; those measurement data were obtained from blood sample.

All statistical analyses were performed using the SAS system for Windows (version 9.4; SAS Inc., Cary, NC). A *p* value < 0.05 was considered statistically significant.

## Results

### Characteristics of study population by HTLV-1 infection status

The characteristics of the study population are shown in Table 1. The proportion of males with HTLV infection was lower than that of non-infected individuals. However, when compared with non-HTLV-1-infected subjects, the HTLV-1-infected subjects exhibited low prevalence in current drinkers and low values of diastolic blood pressure and γ-GTP, but high values in terms of age.

**Table 1:** Characteristics of the study population based on status of HTLV-1 infection

	HTLV-1 infection		*p*
	(−)	(+)	
No. of participants	2409	580	
Men, %	40.2	31.6	< 0.001
Age, years	72.6 ± 7.3	74.7 ± 7.3	< 0.001
Current drinker, %	35.9	29.0	0.002
Current smoker, %	8.3	6.4	0.124
Systolic blood pressure (SBP), mmHg	140 ± 19	140 ± 20	0.936
Diastolic blood pressure (DBP), mmHg	80 ± 12	78 ± 12	0.007
Anti-hypertensive medication, %	39.4	37.8	0.458
Body mass index (BMI), kg/m^2^	23.1 ± 3.4	23.2 ± 3.5	0.268
Triglycerides, mg/dL	104 ± 56	104 ± 57	0.955
HDL-cholesterol, mg/dL	61 ± 15	60 ± 15	0.199
HbA1c, %	5.8 ± 0.6	5.8 ± 0.5	0.581
White blood cell, cells/μL	5532 ± 1465	5558 ± 1463	0.698
γ-Glutamyltransferase (γ-GTP), U/L	21 [15,31]^*1^	19 [15,29]^*1^	0.004^*2^
Platelet, ×10^4^/μL	22.4 ± 5.3	22.5 ± 6.1	0.903

Values, mean ± standard deviation. Regression models for mean values were used for determining the *p*-values

*HDL* high-density lipoprotein

^*1^Values are median [first quartile and third quartile]

^*2^Logarithmic transformation was used for evaluating *p*

### Association between HTLV-1 and hypertension

HTLV-1 infection was significantly associated with hypertension in an inverse manner (Model 1). Furthermore, the same association was observed even after adjusting for known cardiovascular risk factors (model 2) (Table 2).

**Table 2:** Odds ratios (ORs) and 95% confidence intervals (CIs) for hypertension in relation to status of HTLV-1 infection

	HTLV-1 infection		*p*
	(−)	(+)	
Total subjects			
No. of participants	2409	580	
No. of cases (%)	1602 (66.5)	368 (63.4)	
Model 1	1.00	0.79 (0.65, 0.96)	0.020
Model 2	1.00	0.75 (0.62, 0.92)	0.006

*Model 1* adju**s**ted only for sex and age, *Model 2* adjusted further for sex and age, body mass index, triglycerides, HDL-cholesterol, HbA1C, white blood cell**,** and γ-GTP

### Association between platelet and hypertension by HTLV-1 infection status

Although significant positive associations between platelets and hypertension were observed for non-HTLV-1-infected subjects, no significant associations were observed for HTLV-1-infected subjects (Table 3).

**Table 3:** Odds ratios (ORs) and 95% confidence intervals (CIs) for hypertension in relation to platelets by status of HTLV-1 infection

	Platelet tertiles			*p*	1 SD increment of platelets
	T1 (low)	T2 (moderate)	T3 (high)		
HTLV-1 infection (−)					
No. of participants	791	802	816		
No. of cases (%)	492 (62.2)	536 (66.8)	574 (70.3)		
Model 1 (2 categories)	1.00	1.48 (1.23, 1.77)		< 0.001	1.27 (1.16, 1.40)
Model 1 (3 categories)	1.00	1.33 (1.08, 1.64)	1.65 (1.33, 2.04)	< 0.001	
Model 2 (2 categories)	1.00	1.30 (1.07, 1.59)		0.008	1.18 (1.06, 1.30)
Model 2 (3 categories)	1.00	1.23 (0.99, 1.53)	1.40 (1.11, 1.76)	0.004	
HTLV-1 infection (+)					
No. of participants	210	191	179		
No. of cases (%)	137 (65.2)	123 (64.4)	108 (60.3)		
Model 1 (2 categories)	1.00	0.96 (0.67, 1.38)		0.595	0.92 (0.78, 1.07)
Model 1 (3 categories)	1.00	1.03(0.68,1.57)	0.89(0.58,1.36)	0.825	
Model 2 (2 categories)	1.00	0.88 (0.60, 1.29)		0.390	0.88 (0.75, 1.05)
Model 2 (3 categories)	1.00	0.94 (0.61, 1.45)	0.82 (0.52, 1.29)	0.520	

*Model 1* adjusted only for sex and age, *Model 2* adjusted further for sex and age, body mass index, triglycerides, HDL-cholesterol, HbA1C, white blood cell, and γ-GTP. Platelet tertiles: For men < 19.3 × 10^4^/μL for T1 (low), 19.3–23.3 × 10^4^/μL for T2 (moderate), ≥ 23.4 × 10^4^/μL for T3 (high). For women < 20.7 × 10^4^/μL for T1 (low), 20.7–24.8 × 10^4^/μL for T2 (moderate), ≥ 24.9 × 10^4^/μL for T3 (high). The 1 standard deviation (SD) increment of platelet was 5.24 × 10^4^/μL both for men and women

An investigation into the effects of the associations between HTLV-1 infection and 1 SD increment of platelets on hypertension revealed significant interactions (*p* ≤ 0.001 for model 1 and p = 0.001 for model 2).

### Association between HTLV-1 and hypertension by platelet levels

Table 4 shows the association between HTLV-1 and hypertension according to the platelet levels (men, < 19.3 × 10^4^/μL for T1 (low), 19.3–23.3 × 10^4^/μL for T2 (moderate), ≥ 23.4 × 10^4^/μL for T3 (high); women < 20.7 × 10^4^/μL for T1 (low), 20.7–24.8 × 10^4^/μL for T2 (moderate), ≥ 24.9 × 10^4^/μL for T3 (high)). Although no significant associations between HTLV-1 and hypertension were observed for subjects with the lowest platelet levels (the lowest tertile level of platelet), a significant inverse association was observed for high platelet levels (≥ second tertile levels of platelets).

**Table 4:** Odds ratios (ORs) and 95% confidence intervals (CIs) for hypertension in relation to status of HTLV-1 infection by platelet levels

	Platelet levels						Interaction
	The lowest (T1)			Higher (T2 + T3)			
	HTLV-1 infection		*p*	HTLV-1 infection		*p*	
	(−)	(+)		(−)	(+)		
Total subjects							
No. of participants	791	210		1618	370		
No. of cases (%)	492 (62.2)	137 (65.2)		1110 (68.6)	231 (62.4)		
Model 1	1.00	1.06 (0.77, 1.47)	0.721	1.00	0.68 (0.53, 0.87)	0.002	0.037
Model 2	1.00	1.01 (0.72, 1.42)	0.945	1.00	0.64 (0.50, 0.82)	< 0.001	0.004

*Model 1* adju**s**ted only for sex and age, *Model 2* adjusted further for sex and age, body mass index, triglycerides, HDL-cholesterol, HbA1C, white blood cell**,** and γ-GTP. Platelet level: The lowest (T1) is < 19.3 × 10^4^/μL for men and < 20.7 × 10^4^/μL for women. Higher (T2 × T3) is ≥ 19.3 × 10^4^/μL for men and ≥ 20.7 × 10^4^/μL for women

We also found a significant effect of the interaction of two platelet categories (the lowest and the higher (≥ second tertile levels of platelet)) on the association between HTLV-1 and hypertension (*p* = 0.037 for model 1, and *p* = 0.004 for model 2).

### Association between platelet and atherosclerosis by HTLV-1 infectious status among non-hypertensive subjects

Although no significant associations between platelets and atherosclerosis were observed in non-HTLV-1-infected subjects, significant positive associations were observed for HTLV-1-infected subjects (Table 5).

**Table 5:** Odds ratios (ORs) and 95% confidence intervals (CIs) for atherosclerosis in relation to platelets by status of HTLV-1 infection among non-hypertensive subjects

	Platelet tertiles			*p*	1 SD increment of platelets
	T1 (low)	T2 (moderate)	T3 (high)		
HTLV-1 infection (−)					
No. of participants	299	266	242		
No. of cases (%)	80 (26.8)	79 (29.7)	64 (26.4)		
Model 1 (2 categories)	1.00		1.09 (0.76, 1.57)	0.633	1.12 (0.94, 1.34)
Model 1 (3 categories)	1.00	1.33 (0.90, 1.98)	1.26 (0.83, 1.90)	0.253	
Model 2 (2 categories)	1.00		0.97 (0.66, 1.42)	0.615	1.06 (0.88, 1.28)
Model 2 (3 categories)	1.00	1.27 (0.85, 1.91)	1.11 (0.71, 1.71)	0.878	
HTLV-1 infection (+)					
No. of participants	73	68	71		
No. of cases (%)	20 (27.4)	20 (29.4)	32 (45.1)		
Model 1 (2 categories)	1.00		2.36 (1.26, 4.44)	0.008	1.39 (1.04, 1.85)
Model 1 (3 categories)	1.00	1.15 (0.54, 2.46)	2.54 (1.21, 5.31)	0.008	
Model 2 (2 categories)	1.00		2.63 (1.31, 5.29)	0.007	1.44 (1.05, 1.97)
Model 2 (3 categories)	1.00	1.21 (0.55, 2.65)	2.88 (1.30, 6.40)	0.011	

*Model 1* adjusted only for sex and age, *Model 2* adjusted further for sex and age, body mass index, triglycerides, HDL-cholesterol, HbA1C, white blood cell, and γ-GTP. Platelet tertiles: For men < 19.3 × 10^4^/μL for T1 (low), 19.3–23.3 × 10^4^/μL for T2 (moderate), ≥ 23.4 × 10^4^/μL for T3 (high). For women < 20.7 × 10^4^/μL for T1 (low), 20.7–24.8 × 10^4^/μL for T2 (moderate), ≥ 24.9 × 10^4^/μL for T3 (high). The 1 standard deviation (SD) increment of platelet was 5.24 × 10^4^/μL both for men and women

However, an investigation into the effects of the associations between HTLV-1 infection and 1 SD increment of platelet on atherosclerosis showed no significant value of interactions (*p* = 0.192 for model 1 and *p* = 0.179 for model 2).

### Association between HTLV-1 and atherosclerosis by platelet levels among non-hypertensive subjects

Table 6 shows the association between HTLV-1 and atherosclerosis by platelet levels among the non-hypertensive subjects. Although no significant associations between HTLV-1 and hypertension were observed for subjects with a low platelet level (≤ second tertile level of platelet), a significant positive association was observed for subjects with the highest platelet level (the highest tertile levels of platelets).

**Table 6:** Odds ratios (ORs) and 95% confidence intervals (CIs) for atherosclerosis in relation to status of HTLV-1 infection by platelet levels among non-hypertension

	Platelet levels						Interaction
	Lower (T1 + T2)			The highest (T3)			
	HTLV-1 infection		*p*	HTLV-1 infection		*p*	
	(−)	(+)		(−)	(+)		
Total subjects							
No. of participants	299	73		508	139		
No. of cases (%)	80 (26.8)	20 (27.4)		143 (28.1)	52 (37.4)		
Model 1	1.00	0.96 (0.62, 1.49)	0.864	1.00	2.18 (1.20, 3.94)	0.010	0.032
Model 2	1.00	0.89 (0.57, 1.39)	0.612	1.00	2.11 (1.15, 3.86)	0.016	0.035

*Model 1* adjusted only for sex and age, *Model 2* adjusted further for sex and age, body mass index, triglycerides, HDL-cholesterol, HbA1C, white blood cell, and γ-GTP. Platelet level: Lower (T1 × T2) is < 23.4 × 10^4^/μL for men and < 24.9 × 10^4^/μL for women. The highest (T3) is ≥ 23.4 × 10^4^/μL for men and ≥ 24.9 × 10^4^/μL for women

We also found that the interaction of two platelet categories (low and the highest) had a significant effect on the association between HTLV-1 and atherosclerosis.

For sensitivity analysis, we again performed the main analyses using the definition of controlled hypertension and obtained the same associations. We also re-performed the main analyses using drinking status (non-drinker, former drinker, and current drinker) and smoking status (non-smoker, former smoker, and current smoker) as adjusting factors instead of adjusting for γ-GTP and white blood cell counts; importantly, we obtained essentially the same associations.

## Discussion

In terms of the major findings of this study, HTLV-1 infection was found to be inversely associated with hypertension, in particular for subjects with higher platelet levels. Among the subjects without hypertension, HTLV-1 infection was found to be positively associated with atherosclerosis, limited to subjects with the highest platelet levels.

Platelets play an important role in present findings. In conjunction with hematopoietic stem cells (CD34-positive cells), activating platelets are considered to be involved in the initial mechanisms that contribute to endothelial repair, and endothelial injury activates this process [25–27]. These activated platelets induce the differentiation of circulating hematopoietic stem cells into endothelial progenitor [28], mural, and foam cells [29], which are the known sources of atherosclerosis. Since atherosclerosis is the process of aggressive endothelial repair [30], the number of platelets can indicate the level of endothelial repair activity [31]. Therefore, a high level of platelets and the presence of hematopoietic stem cells are mandatory for the development of atherosclerosis [32].

Additionally, these processes also play an important role in the development of angiogenesis [33, 34], which reduces peripheral blood pressure. Since anti-angiogenic therapies induce hypertension [35], characteristics that develop atherosclerosis could have beneficial effects on preventing hypertension through the development of angiogenesis [36].

**Fig. 1:**
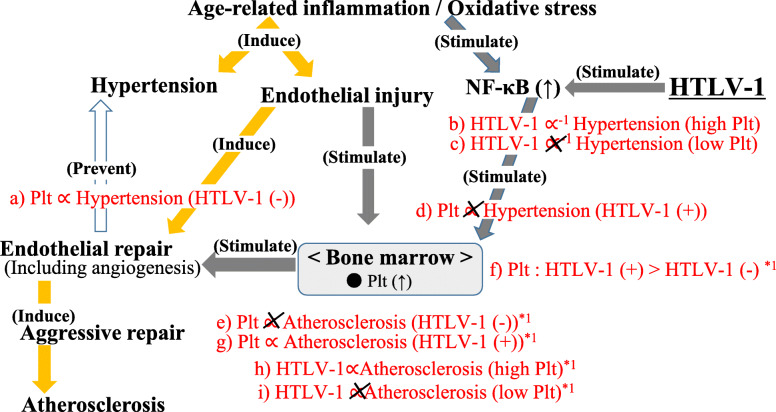
Possible mechanism of endothelial maintenance activity in association with HTLV-1 infection. Plt, platelet. Associations shown in red (**a~i**) were observed in the present study. *1 indicates the associations observed among non-hypertensive subjects. HTLV-1 might activate platelet production and develop not only atherosclerosis but also angiogenesis, which is beneficial for preventing hypertension

The possible mechanisms underlying the present results are summarized in Fig. 1. Our previous study found that platelet levels were positively associated with hypertension [37]. This is compatible with our present study, which showed a positive association between platelets and hypertension among subjects without HTLV-1 infection (Table 3, Fig. 1a). Hypertension is known to induce endothelial injury. Platelets count indicates the level of endothelial repair activity [31]. Therefore, endothelial injury, which is associated with hypertension, may stimulate platelet production, resulting in a positive association between platelet count and hypertension.

However, no significant association was found between platelet and hypertension in subjects with HTLV-1 infection (Table 3, Fig. 1d). HTLV-1 possesses a characteristic of an inflammation inducer since the p40Tax trans-activator of HTLV-1 enhances inflammation [10, 11], possibly by activating the NF-κB pathway [12]. Since the activation of the NF-κB pathway could also promote the production of platelet activation proteins [19], the platelet count in subjects with HTLV-1 infection no longer indicates the magnitude of endothelial injury caused by hypertension.

Furthermore, platelet-rich plasma has been reported to have beneficial effects on angiogenesis [38], which could reduce peripheral blood resistance, the subjects infected with HTLV-1 with a high platelet count may have a beneficial association with hypertension. Therefore, in the present study, we found that HTLV-1 infection was significantly inversely associated with hypertension in subjects with a high platelet count (≥ second tertiles of platelet level) (Table 4, Fig. 1b) but not among subjects with a low platelet count (the lowest tertile of platelet level) (Table 4, Fig. 1c). Of note, for subjects without HTLV-1 infection, endothelial injury, which is associated with hypertension, may stimulate platelet production, resulting in a positive association between platelet counts and hypertension (Table 3, Fig. 1a); conversely, HTLV-1 infected subjects with high platelet numbers may have a beneficial (inverse) association with hypertension, possibly due to the activation of the angiogenesis (Table 4, Fig. 1b, c).

The beneficial association between HTLV-1 infection and hypertension may result in a high chance of progressing angiogenesis associated with developing atherosclerosis. Therefore, the analysis of non-hypertensive subjects revealed that HTLV-1 infection is significantly positively associated with atherosclerosis in subjects with the highest platelet level (the highest tertile of platelet level) (Table 6, Fig. 1h, i).

Furthermore, among the non-hypertensive subjects, compared to subjects without HTLV-1 infection, subjects with HTLV-1 infection showed significantly higher platelet counts (*p* = 0.006); the sex- and age-adjusted values (least mean square ± standard error (SE)) of the platelet count were 21.8 ± 0.2 (× 10^4^/μL) for subjects without HTLV-1 and 23.1 ± 0.4 (× 10^4^/μL) for subjects with HTLV-1, respectively (Fig. 1f). Therefore, among non-hypertensive subjects, HTLV-1 infection may stimulate platelet production resulting in development of atherosclerosis. The present analysis limited to subjects without hypertension showed no significant association between platelet count and atherosclerosis in subjects without HTLV-1 infection (Table 5, Fig. 1e), whereas a significantly positive association was found between platelet count and atherosclerosis in subjects with HTLV-1 infection (Table 5, Fig. 1g).

Conversely, this stimulated platelet production due to HTLV-1 infection can be masked through hypertension-related endothelial injury-induced platelet production. Thereafter, among the hypertensive subjects, no significant difference between without [22.7 ± 0.1 (× 10^4^/μL)] and with [22.2 ± 0.3 (× 10^4^/μL)] HTLV-1 infection was observed (*p* = 0.091).

In terms of the strengths of our study, all of our results can be explained by simple mechanisms. Multi-faceted analyses allowed us to determine the possible mechanisms underlying the present results. Furthermore, this study also indicates the mechanism for the activation of atherosclerosis progression, which could have a beneficial influence on preventing hypertension. This is partly compatible with a previous study, which reported no association between CIMT progression and the risk of a cardiovascular event [39], whereas hypertension is a well-known risk factor of cardiovascular disease [40]. Therefore, active endothelial repair associated with atherosclerosis might have a beneficial association with hypertension [36]. Since atherosclerosis is generally regarded as being strongly associated with hypertension [37], the present findings provide a basis for the development of a novel strategy for the prevention of hypertension.

The potential limitations of this study warrant consideration. First, the activation of the NF-κB protein may play an important role in our results; however, no data concerning NF-κB protein were available. Future studies considering the activity of NF-κB protein will be necessary. The factor that directly inhibits platelet activity and angiogenesis could act as a strong confounding factor in the present analysis as under the influence of these factors; high platelet levels no longer indicate enhanced activity of endothelial repair. However, we found significant associations even without information on these factors. Furthermore, since this was a cross-sectional study, causal relationships could not be established. However, multi-faceted analyses enabled us to determine the possible mechanism underlying our results.

## Conclusion

HTLV-1 infection is inversely associated with hypertension, particularly in subjects with high platelet levels. Among subjects with non-hypertension, HTLV-1 infection is positively associated with atherosclerosis, limited to subjects with the highest platelet level. Endothelial repair, including the development of atherosclerosis, may have an inverse association with hypertension.

## Data Availability

The datasets generated and/or analyzed during the current study are not publicly available due to ethical considerations but are available from the corresponding author on reasonable request.

## Abbreviations

HTLV-1: Human T-cell leukemia virus type 1
SNP: Single nucleotide polymorphism
BRAP: Breast cancer suppressor BRCA1**-**associated protein
OR: Odds ratio
CI: Confidence interval
BMI: Body mass index
HDL: High-density lipoprotein
γ-GTP: γ-Glutamyltransferase
*CIMT*: Carotid intima-media thickness
CLEIA: Chemiluminescent enzyme immunoassay
SD: Standard deviation
SE: Standard error
